# Fracture Behavior of Long Fiber Reinforced Geopolymer Composites at Different Operating Temperatures

**DOI:** 10.3390/ma15020482

**Published:** 2022-01-09

**Authors:** Kinga Korniejenko, Beata Figiela, Celina Ziejewska, Joanna Marczyk, Patrycja Bazan, Marek Hebda, Marta Choińska, Wei-Ting Lin

**Affiliations:** 1 Faculty of Materials Engineering and Physics, Cracow University of Technology, al. Jana Pawła II 37, 31-864 Kraków, Poland; celina.ziejewska@pk.edu.pl (C.Z.); joanna.marczyk@pk.edu.pl (J.M.); patrycja.bazan@pk.edu.pl (P.B.); mhebda@pk.edu.pl (M.H.); 2 Research Institute in Civil and Mechanical Engineering GeM-UMR CNRS 6183, 58, Nantes University—IUT Saint-Nazaire, rue Michel Ange, 44 600 Saint Nazaire, France; marta.choinska@univ-nantes.fr; 3 Department of Civil Engineering, National Ilan University, No. 1, Sec. 1, Shennong Rd., Yilan City 26041, Taiwan; wtlin@niu.edu.tw

**Keywords:** geopolymer composite, fiber reinforcement, long fiber, aramid fiber, carbon fiber, glass fiber

## Abstract

The aim of this article was to analyze the fracture behavior of geopolymer composites based on fly ash or metakaolin with fine aggregate and river sand, with three types of reinforcement: glass, carbon, and aramid fiber, at three different temperatures, approximately: 3 °C, 20 °C, and 50 °C. The temperatures were selected as a future work temperature for composites designed for additive manufacturing technology. The main research method used was bending strength tests in accordance with European standard EN 12390-5. The results showed that the addition of fibers significantly improved the bending strength of all composites. The best results at room temperature were achieved for the metakaolin-based composites and sand reinforced with 2% wt. aramid fiber—17 MPa. The results at 50 °C showed a significant decrease in the bending strength for almost all compositions, which are unexpected results, taking into account the fact that geopolymers are described as materials dedicated to working at high temperatures. The test at low temperature (ca. 3 °C) showed an increase in the bending strength for almost all compositions. The grounds of this type of behavior have not been clearly stated; however, the likely causes of this are discussed.

## 1. Introduction

In 1970, the term “geopolymer” was first used. It was introduced by the French scientist professor Joseph Davidovits for the inorganic, amorphous, synthetic aluminosilicate polymers made from the synthesis of silicon (Si) and aluminum (Al) [1,2]. His research was based on earlier work, conducted since at least 1908, on puculane-activated materials [3,4]. Originally, geopolymers were studied as fire-resistant materials, providing an alternative to thermosetting polymers. In this field, in 1973–1976, the first applications of geopolymers in construction as fire-resistant chipboard consisting of a wooden core covered with two geopolymer coatings was developed [5,6]. During the next years, the research confirmed good fire resistance up to 1000 °C, and in the case of modifications to higher temperatures, it included no emission of toxic fumes during heating [7,8]. Moreover, other research shows the possibility of the production of thermal insulation materials from geopolymers, thanks to their features, such as low thermal conductivity, high thermal stability, non-flammability, possibility of manufacturing using low-cost green technology, and safety for humans [5,9,10,11,12]. Geopolymers also have other advantages, such as long-term durability and resistance in corrosive environments, which could also be beneficial in applications as insulation materials [13,14]. Nowadays, on the market, there are commercial applications for geopolymers where their resistance to high temperature is utilized, for example [8,15]:

- SKOBIFIX 30—geopolymer foam dedicated for heating systems, produced by Skoberne, Pfungstadt, Germany;
- Nu-Core^®^A2FR—fireproof geopolymer composite panels, produced by Nu-core^®^, Canberra, Australia;
- Ino-Flamm^®^—fire resistant geopolymer paint, produced by INOMAT, Neunkirchen, Germany;
- Desil Al—binder systems to the foundry industry, produced by Vodnis Klo, Prague, Czech Republic.

The literature on the subject also shows promising research results with the designed geopolymer composites for high-temperature applications with the addition of different types of fiber reinforcements [7,16]. This kind of addition reduces brittle behavior at high temperatures, splitting in case of fire, and increases mechanical properties, such as bending strength, compared to the pure geopolymer matrix [16,17]. The most popular additions for geopolymers dedicated to high temperatures are steel, carbon, and basalt fibers.

Research using steel fibers was provided by Shaikh and Hosan [18]. They tested geopolymer composites with 0, 0.5, 1, and 1.5% steel fibers under the conditions of alternating between soaking the sample in water and drying it at 100 °C, in 24-h cycles, in an acidic environment (soaking the sample in hydrochloric acid and drying it at 100 °C in 24-h cycles). Then, they research the effect of increased temperature on material strength (cyclic heating of the samples and cooling for 90 days at temperatures: 100 °C, 200 °C, and 800 °C) [18]. Research showed the beneficial effect of steel fibers on the mechanical properties and durability of composites. The highest values were obtained for composites containing 1.5% steel fibers. For the matrix material, which was not influenced by any additional factors, the value was 52.6 MPa, and for composites with a fiber content of 1.5%, 65.4 MPa. For different temperatures, the values of compressive strength tests were as follows: 100 °C—about 48 MPa and 53 MPa, 200 °C—about 41 MPa and 44 MPa, 800 °C—about 17 MPa and 29 MPa. The research showed a significant resistance of composites with steel fibers to environmental conditions, including at elevated temperatures [18].

Research on the high-temperature behavior of geopolymer composites with the addition of short carbon fibers was carried out on a fly ash geopolymer matrix with the addition of short fibers at 0.0, 0.5, 1.0, and 1.5% by weight. The tests were carried out at the following temperatures: 28 °C, 200 °C, 400 °C, 600 °C, and 800 °C [19]. The results of the compressive strength tests at ambient temperature showed an increase in its value for composites containing 1 and 1.5% carbon fiber (ca. 31 and 32 MPa, respectively) and a decrease in properties for a material containing 0.5% fiber (ca. 27 MPa), in relation to the material without additives (ca. 29 MPa) [19]. At temperatures of 200 °C, 400 °C, and 600 °C, the properties of composites increase compared to the corresponding materials tested at an ambient temperature of 28 °C. At 800 °C, there was a decrease in the properties of some composites, while for samples with a content of 0 and 1.5%, there was a slight increase in compressive strength, and for composites with a content of 0.5 and 1.5%, it decreased. The highest value in the tests was achieved by composites with fibers at the temperature of 200 °C: 0.5%, ca. 36 MPa; 1%, ca. 40 MPa; and 1.5%, ca. 36 MPa. The sample without fiber addition obtained the highest compressive strength value at a temperature of 600 °C, approximately 37 MPa [19]. The research confirms the possibility of using composites with the addition of carbon fiber in applications for high temperatures [19].

Other studies on the addition of short carbon fibers, taking into account the influence of temperature on the mechanical properties of composites, were carried out in a matrix based on a mixture of metakaolin and fly ash [20]. Short carbon fibers were added to the composites in the following proportions: 0.0, 0.5, 1.0, and 2% by weight. Samples were tested after 7 days at room temperature and 500 °C [20]. The results of the compressive strength at ambient temperature showed a decrease in the value from about 50 MPa for the material without the addition of fibers to approximately 45 MPa for the material with the 2% addition of carbon fibers. However, at a temperature of 500 °C, fiber material with the addition of fibers was more durable and reached approximately 5 MPa for fiber-based composites, compared to approximately 2 MPa for fiber material without fiber addition [20]. The bending strength increased with the addition of fibers, both for the ambient temperature and for 500 °C. For the material without fiber addition, it was approximately 5.5 MPa and below 0.1 MPa, and for composites with a 2% addition of carbon fibers, it was 15 MPa and 1 MPa [20].

Research was also conducted on the addition of carbon microfibers and nanotubes to geopolymer composites. Research on the addition of microfibers (fibers with a length of approximately 100 μm) was carried out in the ratio of 0, 5, 10, and 15% by weight to the metakaolin-based geopolymer matrix [21]. The tests were carried out after 28 days at temperatures of 30 °C, 200 °C, 400 °C, and 800 °C. The highest values for the temperatures of 30 °C and 200 °C were obtained for the 10% addition of carbon microfiber, while the values for 200 °C were higher than for the temperature of 30 °C. They were 44.2 MPa at 30 °C for the material with 10% microfiber addition and 28.4 MPa for a matrix material, while at 200 °C, they were 48.8 MPa for the material with microfibers and 36.6 MPa for the matrix material. The highest values were obtained for temperatures of 400 °C and 800 °C for 15% fiber addition (33.5 MPa and 24 MPa, respectively) for the same temperatures, the compressive strength of fiber material without the addition of fibers was 14.8 MPa and 11.2 MPa [21].

Research conducted for geopolymers reinforced with basalt fiber also showed increased resistance to elevated temperatures [19,22]. Shaikh and Haque [19] conducted research on a fly ash geopolymer matrix with the addition of basalt fiber in the amounts of 0, 0.5, 1, and 1.5% by weight. The tests were carried out at the following temperatures: 28 °C, 200 °C, 400 °C, 600 °C, and 800 °C [19]. The results of the compressive strength tests at ambient temperature showed an increase in mechanical properties for samples with short fibers compared to the material without additives; the highest value was achieved for a 1% addition of basalt fiber and was ca. 36 MPa (matrix material, ca. 29 MPa) [19]. At temperatures of 200 °C, 400 °C, and 600 °C, the properties of composites increased compared to the same materials tested at an ambient temperature of 28 °C. At a temperature of 800 °C, there was a decrease in the strength properties of the material. The highest strength properties were characteristic of the material at 400 °C—the value obtained in the tests for composites with a 0.5 and 1% addition of basalt fiber was approximately 45 MPa, and for 1.5% of the fiber content and the control sample, it was > 35 MPa [19].

Tests with the addition of basalt microfibers were performed in compressive strength at ambient (30 °C) and elevated temperatures (200 °C, 400 °C, and 800 °C) [22]. The research was carried out on a metakaolin matrix reinforced with basalt microfibers up to 10 µm in size, 5, 10, and 15% by weight of microfibers was applied [22]. For the temperature of 30 °C, the composite with 15% microfiber addition showed the highest strength properties. Its compressive strength was 38.10 MPa, compared to 28.43 MPa for the material of the geopolymer matrix alone. Tests at elevated temperatures also showed an increase in the compressive strength of all samples tested at 200 °C, and then a decrease below the base strength at higher temperatures. The highest values at a temperature of 200 °C were also shown by the composite with 15% microfiber addition; the strength was 43.85 MPa, compared to the matrix material (36.61 MPa) at the same temperature. At temperatures of 400 °C and 800 °C, the highest strengths were achieved by the composite with 10% microfiber weight by weight; it was 23.13 MPa and 16.08 MPa, respectively, for comparison, the matrix material at these temperatures reached 14.85 MPa and 11.23 MPa, respectively [22].

Celik et al. [23] compared the behavior of geopolymer composites of basalt fibers with other man-made fibers, such as PA and PVA. Composites with basalt fiber achieved higher values in terms of bending strength than for other compared additives, i.e., polyolefin, PA, and PVA fibers, and in the case of compressive strength, they achieved the second result (composites with PVA fibers added slightly better results) [23]. In addition, as part of the research, the behavior of geopolymeric materials at high temperatures, i.e., 300 °C, 600 °C, and 900 °C, was tested. A significant deterioration of mechanical properties was found at temperatures of 600 °C and 900 °C; however, fiber samples with the addition of fibers were still characterized by values higher than those of the geopolymer matrix material itself [23].

Some research was also conducted on natural fibers such as cotton [24,25], sisal [26], and jute [26,27]. The conducted research also found the thermal stability of these types of composites. Cotton fiber composites have stable properties at elevated temperatures due to the geopolymer matrix [24,25]. Furthermore, fire resistance tests for boards made of natural fiber reinforced geopolymer show that such elements have significant insulating properties and constitute an effective barrier to high temperature. The temperature reduction was ca. 80–90% near the flame [26]. This confirms the fire-resistant properties of geopolymers and additionally indicates that they provide protection for flammable plant fibers [26]. An additional benefit is the change in the nature of the material’s behavior during fracture. The addition of fibers allows the inhibition of typically brittle cracking, which occurs for geopolymer materials at elevated temperatures (tests were carried out at up to 250 °C) [26,27].

Much less research has been carried out on geopolymers at reduced or variable temperatures [28,29]. Bindiganavile et al. [28] conducted research using PP and steel fiber reinforcement based on a geopolymer matrix obtained from fly ash of class C; 0.5 and 1.0% fiber addition per volume were applied. The tests were carried out at temperatures ranging from −30 °C to 300 °C [27]. The test results showed a decrease in the mechanical properties of the composites with temperature. The best results, both in terms of compressive strength and bending strength, were for negative temperatures [28]. For compressive strength, the best results were obtained for samples without fiber addition (approximately 45 MPa; for comparison with 0.5% samples, approximately 38 MPa; and 1%, 30 MPa), and for bending strength, similar to tensile strength, samples with 1% fiber addition—9 MPa (samples without reinforcement addition and with 0.5% addition were <7.2 MPa) [28].

The aim of the research is to analyze the behavior of geopolymer composites based on fly ash or metakaolin with fine aggregate and river sand, with three types of reinforcement: glass, carbon, and aramid fiber, in three different temperatures ca. 3 °C, 20 °C, and 50 °C. The results obtained could be useful in designing products for the construction industry using geopolymer composites. The literature shows a low amount of research provided for geopolymers reinforced by long fibers at elevated and lowered temperatures, including only a few investigations for typical operating temperatures. Because of that, the new work in this area has a significant impact on creating new knowledge about the behavior of these composites. Research in the literature also does not show any information on long fiber-reinforced geopolymer composites reinforced in glass fiber, carbon fiber, and aramid fiber for different temperatures. These types of composites were investigated for the first time for specific operating temperatures.

## 2. Materials and Methods

### 2.1. Materials

**Figure 1:**
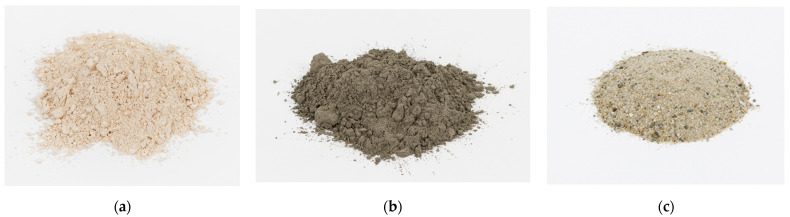
Used raw materials for geopolymer mortar: (**a**) metakaolin; (**b**) fly ash; (**c**) river sand (fine aggregate).

Samples were made with the use of two base materials: metakaolin (Figure 1a) and fly ash from the Skawina Combined Heat and Power Plant (Skawina, Malopolskie, Poland)—Figure 1b, and fine aggregate (Figure 1a). Fine aggregate river sand (Świętochłowice, Poland) was applied. The particle size distribution for the sand showed that most of the particles were between 0.10 and 0.90 mm (approximately 80%) [30].

Metakaolin from the Czech Republic (Keramost, Kadaň, Czech Republic) had the following oxide composition: SiO_2_—53.01%, Al_2_O_3_—41.54%, Fe_2_O_3_—1.34%, Na_2_O—0.82%, TiO_2_—0.74%, K_2_O—0.71%, MgO—0.38% and CaO—0.27%. The used fly ash had an oxide composition typical for class F, including: SiO_2_—55.9%, Al_2_O_3_—23.49%, Fe_2_O_3_—5.92%, CaO—2.72%, K_2_O—3.55%, MgO—2.61%, TiO_2_—1.09% and Na_2_O 0.59% [31].

**Figure 2:**
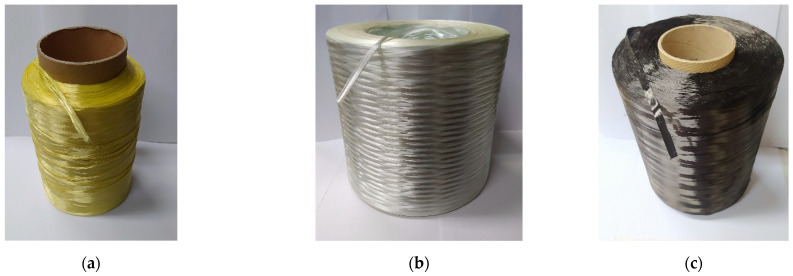
Long fiber (roving): (**a**) aramid fiber; (**b**) glass fiber; (**c**) carbon fibre.

The three types of fibers were applied as reinforcement: aramid, glass-type E, and carbon fiber (P.P.H.U. SURFPOL Jacek Woźniak, Rawa Mazowiecka, Poland), Figure 2. The fibers were selected because of their availability and high mechanical properties. The fibers were cut to a length relevant to the length of the samples, approximately 200 mm. The used roving has 800 tex in each case. The single fiber in the roving had the following diameters: 8 μm carbon fiber, 10 μm aramid fiber, and 10 μm fiberglass.

As an activator, sodium hydroxide (NaOH) (PCC Rokita SA, Brzeg Dolny, Poland) mixed with sodium silicate (Na_2_SiO_3_) (STANLAB, Gliwice, Poland) was applied. The solution was obtained from technical sodium hydroxide flakes, an aqueous solution of sodium silicate (type R-145, density 1.45 g/cm^3^), and tap water. The ratio of sodium base to water glass was 1:2.5. The solution was thoroughly mixed and allowed to equilibrate to a constant concentration and temperature before combining with the solids of the mixture (24 h).

### 2.2. Sample Preparation

**Figure 3:**
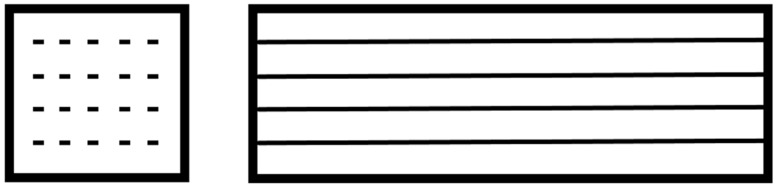
Fiber distribution in the cross-section of the sample and distribution along the sample.

River sand as a fine-grained aggregate was added to the base materials (metakaolin or fly ash) in a 1:1 ratio. The dry ingredients were then mixed for 5 min in a low-speed mixer (Geolab, Warsaw, Poland). Then, the previously prepared activator was added, and the process of mixing was continued for 15 min. After this time, the obtained masses were transferred to a set of prismatic forms (Cracow University of Technology, Cracow, Poland) and combined with the fiber roving. For long fibers (roving), we poured part of the geopolymer mass into the mold, placed the reinforcement, and then covered the rest of the geopolymer mass with the reinforcement (Figure 3).

The samples were prepared with different types of long fibers and their different percentages (Table 1). Additionally, the reference samples were prepared based on metakaolin and fly ash without the addition of fibers. The percentage selection of fibers was based on the previous research, including the best results [32]. The previous results showed that the highest mechanical properties values were obtained in the 2% addition of aramid fibers to the metakaolin matrix. The other samples for comparison were based on different fibers as well as different matrices and different percentages of fibers [32].

**Figure 4:**
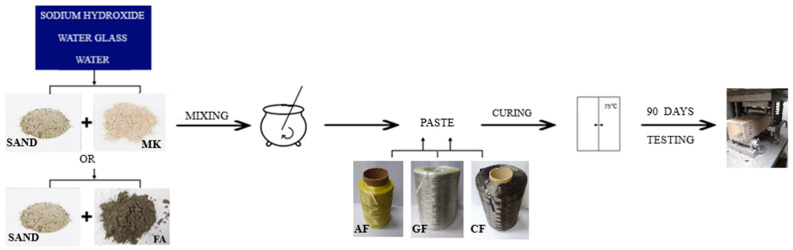
Scheme of sample preparation.

In the next step, the prepared pastes were placed on a vibrating table (Cracow University of Technology, Cracow, Poland) in molds to remove air bubbles. The mold sets were then cured for 24 h at a temperature of 75 °C in a SLW 750 STD laboratory dryer (Pol-Eko-Aparatura, Wodzisław Śląski, Poland). They were covered with foil to avoid rapid water reduction. After 24 h, the samples were cooled to room temperature and demolded. The disassembled samples were stored for 90 days (the time used for the full maturation of composites based on traditional cements). Seasoning was carried out under laboratory conditions. The most important steps of the sample preparation are presented in Figure 4.

### 2.3. Methods

Before bending strength tests were performed, the densities of the samples were determined using the geometric method. They were determined as the average of the measurements for five samples. The dimensions of the samples were measured with an electronic caliper (OVIBELL GmbH and Co. KG, Mülheim an der Ruhr, Germany) with a measuring accuracy of 0.01 mm. Then, they were weighed on a laboratory precise analytical balance (maximum load: 200/2000 g; reading accuracy: 0.001/0.01 g) from RADWAG PS200/2000R2 (RADWAG Wagi Elektroniczne, Radom, Poland). The calculations were made for solid, nonporous materials. Additionally, for the obtained results, the standard deviation was calculated.

The bending strengths of the composites were tested according to PN-EN 12390-5, 2019-08 standard, Concrete tests, Part 5: Bending strength. The test was carried out by three-point bending, that is, a concentrated load was applied on the upper edge of each sample. The measurements were made with a MATEST 3000 kN (Matest, Treviolo, Italy). For the investigation, prismatic samples, with dimensions: 50 × 50 × 200 mm, were prepared. The length between the support points was 150 mm. The tests were based on standards for testing concrete due to the lack of a standard dedicated to geopolymer materials, and the similar nature of the geopolymer composite, as well as the similar nature of the products, especially in the construction industry. Currently, no standards have been developed that are dedicated to the testing of geopolymeric materials. The research was conducted on samples seasoned for 90 days at ambient temperature. Each geopolymer composite was tested on 3 samples at each temperature (min. 9 for one composition). Firstly, the loose debris was removed from the samples to ensure proper contact with the support points (rollers). Then, the samples were placed and centered in the testing machine. The load direction was perpendicular to the direction in which the samples were formed. Next, the constant load speed of 0.05 N/mm^2^∙s was assumed, and the load was increased continuously until the maximum value was reached. Finally, the bending strength was determined from the following Formula (1):

(1) $$f_{cf}=\frac{3\cdot F\cdot I}{2\cdot d_{1}\cdot d_{2}{}^{2}}\;$$

where $f_{cf}$ is the bending strength [MPa], F is the maximum load [N], I is the spacing of the support rollers [mm]$,\;\mathrm{and}\;d_{1},\;d_{2}$ are the transverse dimensions of the sample [mm].

The measurements were made at three temperatures: ambient (approximately 20 °C), lowered (approximately 3 °C), and elevated (approximately 50 °C) temperature. The temperatures were selected according to the predicted temperature of the work-designed materials. The measurement of the temperature—change in thermal radiation—was performed on the surface of the sample using a FLIR thermal imaging camera (Teledyne FLIR LLC, Thousand Oaks, CA, USA) with a field of view (FOV) ≥ 38°, thermal sensitivity < 70 mK, measured infrared wavelength range in the range of 7–14 µm, and pixel size < 15 µm.

## 3. Results

### 3.1. Density

**Figure 5:**
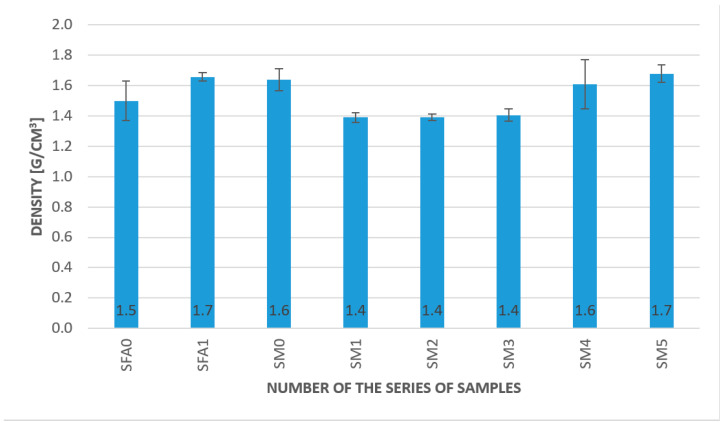
Density test result.

Density changes were not significant, taking into account the fiber addition. These results are presented in Figure 5.

The density values were between 1.4 and 1.7 g/cm^3^. The results for the geopolymers based on metakaolin and fly were comparable. The addition of fibers had a slight influence on the density. Taking into consideration the density of the fibers, it should decrease the density of the whole composition. In the case of applied fibers, all of them had a lower density than the geopolymer matrix [32]. The measurements showed that the other factors, for example, additional voids in the materials created by fiber additions, had a stronger influence on the density than the addition of the fibers between 0.5 and 2.0% by weight.

### 3.2. Bending Strength—Ambient Temperature

**Figure 6:**
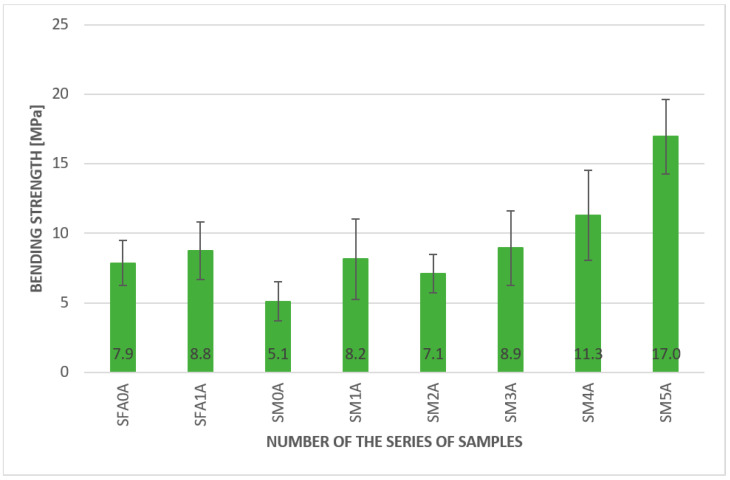
Results of the bending strength test for geopolymer composites at ambient temperature.

The results of the bending strength under ambient temperature are presented in Figure 6. The measurements were made under laboratory conditions, and the ambient temperature was approximately 20 °C.

The samples with fibers in the case of the geopolymer matrix based on metakaolin as well as on fly ash showed higher values of bending strength than the pure matrix. The best results were achieved with the 2.0% aramid fiber addition; it was 17 MPa, compared to only 5.1 MPa, for a plain metakaolin matrix. In addition, the lowest amount of aramid fibers improved the bending strength. The bending strength for the composites with 0.5% and 1.0% of aramid fibers by weight were 8.9 MPa and 11.3 MPa, respectively. A similar tendency was also presented for fly-ash-based geopolymers; however, the improvement was not as significant. The values were 7.9 MPa for the plain matrix and 8.8 MPa for a 2% addition of long aramid fibers. The other fibers, such as glass and carbon fibers, were also investigated in the metakaolin matrix, and they increased the value of the bending strength. The obtained results showed 8.2 MPa for a 2.0% addition of glass fibers and 7.1 MPa for a 2.0% addition of carbon fibers. The results confirm previous research work conducted for these materials [32]. However, it is worthwhile to notice that the differences between the 2% addition of different fibers were not statistically significant when the standard deviation was taken into consideration.

### 3.3. Bending Strength—High Temperature

**Figure 7:**
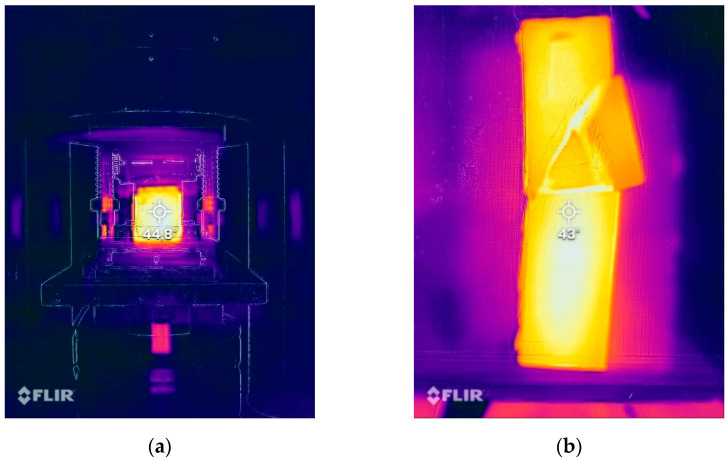
Measurement of change in thermal radiation made using an FLIR thermal imaging camera:=. (**a**) Sample during the bending test. (**b**) Sample after the bending test.

The samples before measurement were stored in a laboratory oven for 24 h. The temperature in the oven was approximately 60 °C. During the test, it was usually between 45 and 50 °C; for the temperature control, a thermal camera was used (Teledyne FLIR LLC, Thousand Oaks, CA, USA). Typical temperature measurements during the bending strength test are presented in Figure 7.

**Figure 8:**
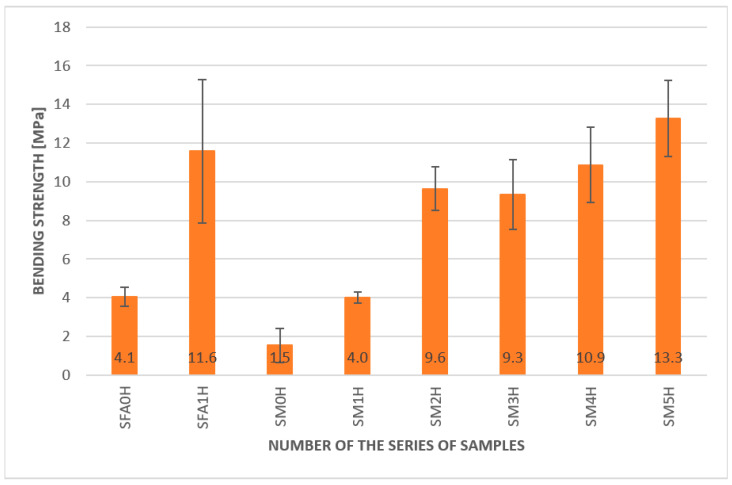
Results of the bending strength test for geopolymer composites at elevated temperature.

The results of the bending strength under elevated temperatures are presented in Figure 8.

The overall tendency of material behavior was similar to the results obtained for the ambient temperature. The fiber additions increased the bending strength of the composites compared to the plain matrix. The best values were obtained for the metakaolin-based geopolymer based on metakaolin with 2.0% addition of aramid fibers (13.3 MPa). The lowest value of the bending strength had a plain metakaolin matrix, only 1.5 MPa. The least amount of aramid reinforcement also significantly improved the bending strength. The values were 9.3 MPa and 10.9 MPa for 0.5% and 1% of aramid fiber addition, respectively. Promising reinforcement was also provided by the carbon fibers. The obtained value for the 2.0% addition of this fiber was 9.6 MPa. In comparison with the composite with the same amount of glass fibers, this value was more than two times higher.

Significant improvement was also observed in the samples based on the fly ash matrix. It was 4.2 MPa for the plain matrix and 11.6 MPa for the composite with a 2.0% aramid fiber addition. It is worthwhile to notice the high value of the standard deviation (error bars) for this sample, which shows a high score spread between particular samples.

### 3.4. Bending Strength—Low Temperature

**Figure 9:**
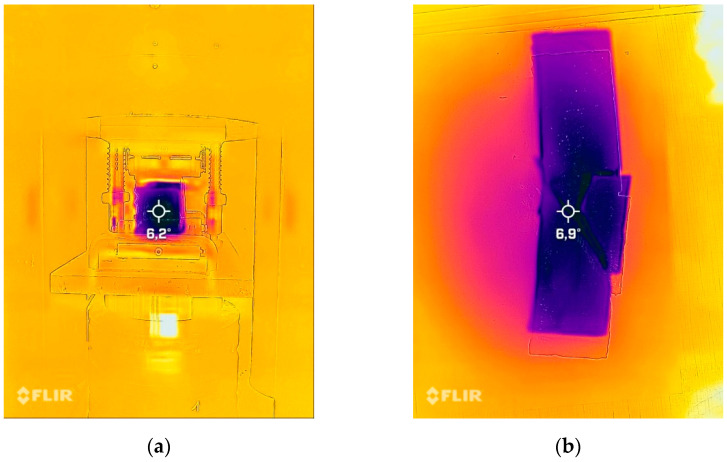
Measurement of change in thermal radiation made using an FLIR thermal imaging camera. (**a**) Sample during the bending test. (**b**) Sample after the bending test.

The samples before the measurement were stored in the refrigerator for 24 h. The temperature in the refrigerator was ca. 3 °C, during the test, it was usually between 5 and 7 °C; for the temperature control, a thermal camera was used. Typical temperature measurements during the bending strength test are presented in Figure 9.

**Figure 10:**
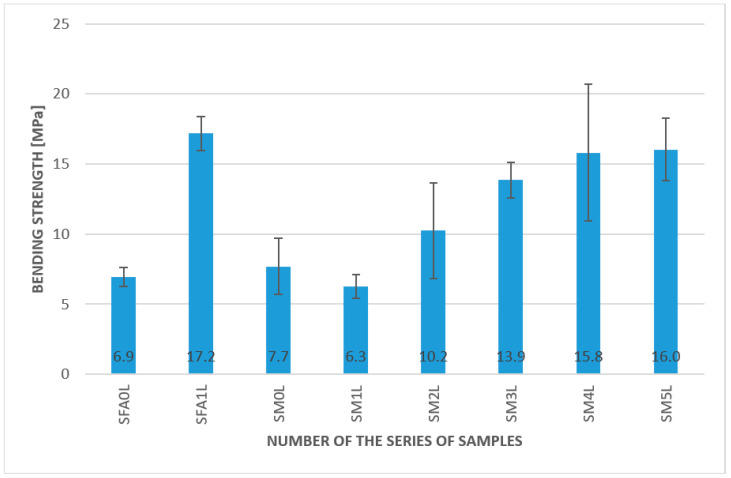
Results of the bending strength test for geopolymer composites with a lowered temperature.

The results of the bending strength at a lower temperature are presented in Figure 10.

In the case of lowered temperatures, the results obtained are slightly different from those for ambient and elevated temperatures. The results show that the glass fibers in these temperatures no longer have a function of reinforcement. The obtained results, in this case, were lower than for a plain geopolymer matrix, 6.3 MPa, and 7.7 MPa, respectively. The other fiber additions reinforced the geopolymer matrix. The best result was obtained for the fly ash-based matrix with a 2.0% aramid fiber addition; it was 17. MPa, compared to 6.9 MPa for the pure fly ash-based matrix. In the case of metakaolin-based composites, the best result was also obtained for the 2.0% addition of aramid fibers; it was 16.0 MPa. Slightly lower results were found for the 1.0% aramid fiber addition (15.8 MPa). For this result, it is important to stress the high score spread between the particular samples. In this case, the samples had a large variation in obtained results, which is reflected in the standard deviation value; because of this, the difference between the 1% and 2% addition of the aramid fibers was not statistically significant. The addition of 0.5% aramid fibers also significantly influenced the obtained value and increased the bending strength to 13.9 MPa. The lowest influence was an addition of 2.0% carbon fiber—the composite with these fibers had 10.2 MPa bending strength.

### 3.5. Study of the Fracture Mechanism

**Figure 11:**
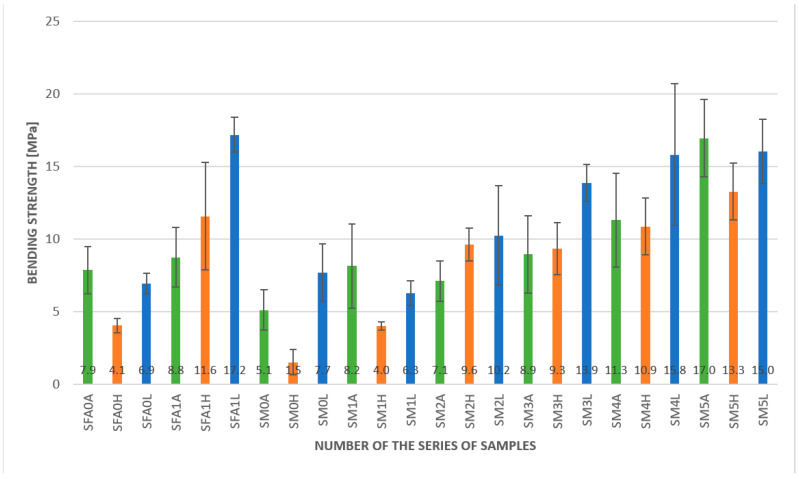
Results of the bending strength test for geopolymer composites—comparison at different temperatures.

In Figure 11 and Table 2, the results of the bending strength obtained for different temperatures are compared.

Table 2 also shows the percentage of changes in elevated and lowered temperatures compared to those obtained at ambient temperature. The decreasing values are marked in red color.

The changes in elevated temperatures show an overall tendency of decreasing mechanical properties (bending strength). Only in three cases did the bending strength increase with increasing temperature, and this value was between 10 and 30%. Taking into consideration the trends that present the geopolymers as a material dedicated to high temperature, did we find these results to be against the literature. However, most of the research is focused on the application of this material in temperatures above 100 °C [33,34].

The changes in lowered temperature showed a reverse tendency to those in elevated temperatures. Most of the investigated compositions obtained better results than at ambient temperature. Only three of them showed a slight decrease in mechanical properties. The increase in mechanical properties in lowered temperature treatments was between 40 and 90%. Additionally, it is worthwhile to notice that for all samples, the obtained results of bending strength were higher for the lower temperatures. The improvement was between approximately 6% for the metakaolin-based geopolymer composite with a 2.0% addition of carbon fibers and more than four times for the plain metakaolin-based matrix. For most samples, this change was between 40 and 80%, showing that the geopolymer composite could be a good material for application at lower temperatures.

The three compositions show that the increased bending strength was independent of temperature. These were the fly ash-based geopolymer with a 2.0% addition of aramid fibers, a metakaolin-based geopolymer with a 2.0% addition of carbon fibers, and a metakaolin-based geopolymer with a 0.5% addition of aramid fibers. Among them, the highest values were obtained for the fly ash-based geopolymer with a 2.0% addition of aramid fibers; this seems to be a promising composite for applications in lower temperatures. The results obtained showed the highest values obtained for the metakaolin composite based on metakaolin with a 2.0% addition of aramid fibers. Even if the value for bending strength for this composition in elevated and lowered temperatures decreased compared to the obtained value at ambient temperature, it was still higher than for the other compositions.

**Figure 12:**
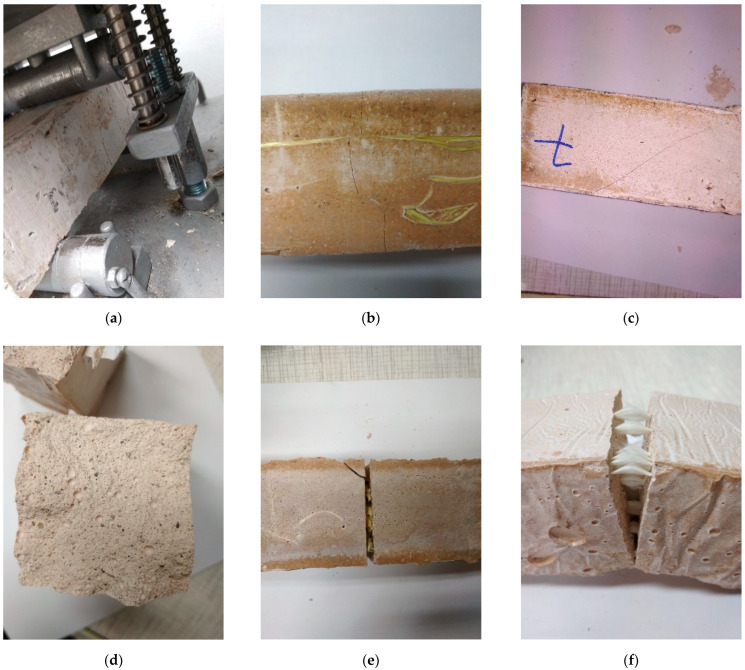
Study of the fracture behavior. (**a**) Sample during the bending test. (**b**) Sample after the bending test—typical mechanism of cracking propagation (in the middle of the sample). (**c**) Sample after the bending test—first crack appears in the middle, and the second crack appears near the edge. (**d**) Sample after the bending test—brittle behavior of the sample without reinforcement. (**e**) Sample after the bending test—more ductile behavior of the sample with aramid fibers. (**f**) Sample after the bending test—more ductile behavior of the sample with glass fibers.

The photographic material helps to analyze the fracture behavior (Figure 12).

The measurements of the values obtained in Figure 11 were made for the first crack. Most of the investigated samples in all temperatures had similar behavior during the bending tests. The typical mechanism of cracking propagation was one single crack in the middle of the sample (Figure 12b). This behavior was observed for approximately 75% of all samples investigated. For 25% of the investigated samples, the first crack appeared in the middle; but in a short time, the next crack appeared near the edge (Figure 12c). This behavior was observed mainly for samples with fibers and was probably caused by stopping the first crack at the appearance of the fiber while accumulating the stress in the other area of the sample where the crack could propagate easier.

During the bending test, the samples without the fibers showed brittle behavior, and at the end of the test, they lost their coherence (Figure 12d). Unlike them, the samples with fibers showed more ductile behavior and left their coherence after the test (Figure 12b,c). For selected samples, the test was applied for a second time to observe the fiber behavior in the material. During the second test, samples usually achieved higher values than in the moment of braking during the first test. The samples that were bending again showed that the fibers have an important role in material coherence. When the gap enlarged itself, the forces were taken over on the fibers. The fibers were lengthened, but they did not lose coherence with the matrix. The pulling out of the fibers was not observed. After the force was released, the gap decreased. In this time, the fibers did not return to the previous form by creating ‘fluffs’ inside the gap (Figure 12e). This behavior was clearly visible for aramid and carbon fibers. The behavior of the fiberglass-reinforced samples was more ductile compared to the samples without reinforcement, but the fibers showed a tendency to break partially during the bending test (Figure 12f). This type of behavior was not related to the temperatures.

## 4. Discussion

The results of the density changes presented in this article align with other research [35,36]. They do not show any significant changes, taking into account the fiber addition. The values obtained are in the range of 1.4 to 1.70 g/cm^3^, which is typical for geopolymer composites [35,36].

The results obtained for different operating temperatures are not in line with the tendency to present geopolymers as a material dedicated to high-temperature and fire-resistant applications [34,37]. The literature shows a decrease in mechanical properties in these materials, but it is usually at the temperature of ca. 800 °C [38]. At temperatures up to 100 °C, the mechanical properties usually slightly increase for both types of matrix fly ash and the metakaolin-based material [38]. The decrease in mechanical properties at high temperatures was explained by temperature-induced thermal deformations and mass loss [38]. At the microstructural level, it was associated with water loss and deterioration bonding between the aggregate and the matrix, but this behavior was observed at significantly higher temperatures, such as 400 °C, 600 °C, and 800 °C [39,40,41,42]. This behavior cannot be explained by the loss of the properties of the fibers used because all of them are resistant to much higher temperatures.

The investigation did not show the reasons for the strength loss. For this range of temperatures, the physical changes or chemical changes have no place for geopolymers nor for used fibers [43,44]. Moreover, the changes in the surface of the samples, such as cracking, were not observed. The sample did not change in dimension with changes in temperature. The most probable reason seems to be moisture loss, but more precise measurements will be required because the laboratory weight used did not confirm significant weight changes. These changes have a place for the composites with fibers as well as without fibers because the fiber influence for the behavior does not seem to be significant.

The behavior under lowered temperatures was in agreement with the available literature; however, there are few articles dedicated to this kind of investigation on geopolymers [27,28,45]. Moreover, the majority of this research did not directly investigate the mechanical behaviors in low temperatures but rather focused on different tests, such as freeze-thaw, and the material properties in low temperatures are described indirectly [28,45]. Comparisons between lower and higher temperatures suggest that the geopolymer composite could be a good material for application at lower temperatures. This topic is worth more detailed future investigations, including a comparison with traditional concrete and investigation in extremely low temperatures.

## 5. Conclusions

The aim of the research presented in the article was to analyze the behavior of geopolymer composites based on fly ash or metakaolin with fine aggregate and river sand, with three types of reinforcements: glass, carbon, and aramid fiber, in three different temperatures, approximately 3 °C, 20 °C, and 50 °C. The results obtained show the following.

- Density changes are not significant, taking into account fiber addition. This value is comparable to the standard geopolymer created using sand as a fine aggregate, where the density value was usually between 1.4 and 1.70 g/cm^3^.
- The samples with fibers in the case of the geopolymer matrix based on metakaolin and fly ash showed higher values of bending strength than the pure matrix. The best results were achieved for the 2.0% addition of aramid fibers. The overall tendency of material behavior in the temperature of approximately 50 °C was similar to results obtained for ambient temperature. The best result was obtained for the geopolymer based on metakaolin with a 2.0% addition of aramid fibers—13.3 MPa.
- The best result was obtained for the fly ash-based matrix with a 2.0% aramid fiber addition; it was 17 MPa, compared to 6.9 MPa for the pure fly ash-based matrix at ambient temperature.
- It was a fly ash-based geopolymer with a 2.0% addition of aramid fibers, a metakaolin-based geopolymer with a 2.0% addition of carbon fibers, and a metakaolin-based geopolymer with a 0.5% addition of aramid fibers. Among them, the highest values were obtained for the fly ash-based geopolymer with a 2.0% addition of aramid fibers, which seems to be a promising composite for applications in lower temperatures.
- Comparison of the results obtained in lowered temperature, approximately 3 °C, to the results obtained in the temperature of approximately 50 °C, showed that in all samples, the values of bending strength were higher in lower temperature. For most samples, this change was between 40 and 80%.
- The results obtained showed the highest values obtained for the metakaolin composite based on metakaolin with a 2.0% addition of aramid fibers. Even if the value for bending strength for this composition in elevated and lowered temperatures decreased compared to the obtained value in ambient temperature, it was still higher than for other compositions.

## Figures and Tables

**Table 1:** Compositions of prepared samples.

Sample Designation	Matrix	Reinforcement
SFA0	Fly ash and sand	-
SFA1	Fly ash and sand	Aramid fiber 2.0% wt.
SM0	Metakaolin and sand	-
SM1	Metakaolin and sand	Fiberglass 2.0% wt.
SM2	Metakaolin and sand	Carbon fiber 2.0% wt.
SM3	Metakaolin and sand	Aramid fiber 0.5% wt.
SM4	Metakaolin and sand	Aramid fiber 1.0% wt.
SM5	Metakaolin and sand	Aramid fiber 2.0% wt.

**Table 2:** Compositions of prepared samples.

Sample	Ambient Temperature [MPa]	High Temperature [MPa]	% Change Compare to Ambient Temperature	Low Temperature [MPa]	% Change Compare to Ambient Temperature
SFA0	7.9	4.1	51.90	6.9	87.34
SFA1	8.8	11.6	131.82	17.2	195.45
SM0	5.1	1.5	29.41	7.7	150.98
SM1	8.2	4.0	48.78	6.8	82.93
SM2	7.1	9.6	135.21	10.2	143.66
SM3	8.9	9.8	110.11	13.9	156.18
SM4	11.3	10.9	96.46	15.8	139.82
SM5	17.0	13.3	78.24	16.0	94.12

## Data Availability

Data sharing is not applicable.
